# miR-223 and Chromogranin A Affect Inflammatory Immune Cell Activation in Liver Metastasis of Neuroendocrine Neoplasms

**DOI:** 10.3390/cells14020111

**Published:** 2025-01-14

**Authors:** Lukas Geisler, Katharina Detjen, Teresa Hellberg, Marlene Kohlhepp, Carsten Grötzinger, Jana Knorr, Ines Eichhorn, Raphael Mohr, Theresa Holtmann, Bertram Wiedenmann, Frank Tacke, Christoph Roderburg, Alexander Wree

**Affiliations:** 1 Department of Hepatology and Gastroenterology, Charité University Medicine Berlin, 13353 Berlin, Germany; 2 Department of Biology, Humboldt University of Berlin, 10099 Berlin, Germany; 3 Department of Gastroenterology, Hepatology and Infectious Diseases, University Hospital Düsseldorf, 40225 Düsseldorf, Germany

**Keywords:** neuroendocrine neoplasm, miR-223, Nlrp3, neutrophil

## Abstract

Neuroendocrine neoplasms (NENs) are a diverse group originating from endocrine cells/their precursors in pancreas, small intestine, or lung. The key serum marker is chromogranin A (CgA). While commonly elevated in patients with NEN, its prognostic value is still under discussion. Secretion/posttranslational proteolytic cleavage of CgA results in multiple bioactive fragments, which are essential regulators of the cardiovascular and immune system. miR-223, regulator of Nrlp3 inflammasome and neutrophil activation, was recently found to have decreased in patients with NEN. We performed flow cytometry of circulating neutrophils in a patient cohort (n = 10) with NEN, microdissection and histology of tumor tissue. Subsequently, in vitro transfections using the well-established human pancreatic NEN cell line (BON), and co-culture experiments with primary macrophages and neutrophils were performed. Serum miR-223 in patients correlated with the expression of the neutrophil activation marker CD15 in circulating cells. Neutrophilic CD62L/CD63 showed good discrimination compared to healthy controls. Immune cell-derived miR-155, miR-193 and miR-223 colocalize with neutrophil in the extra-tumoral tissue alongside Nlrp3-associated caspase-1 activation. miR-223 knockdown in BON decreased the CgA intracellularly, increased in cellular granularity and caspase-1 activation. Plasmin inhibitor a2-aP reverted those effects. Western Blot showed fragmented CgA following miR-223 knockdown, which altered the inflammatory potential of neutrophils. Our data hence provide initial insights into an immunoregulatory mechanism via miR-223 and CgA in NEN cells, as regulation of miR-223 in NEN may affect tumor-associated inflammation.

## 1. Introduction

Neuroendocrine neoplasm (NEN) is a group of neoplasms presumably emerging from non-neoplastic neuroendocrine, secretory cells or their precursors [1,2]. The group of tumors is highly diverse, and they may secrete a plethora of proteins or peptides, some of which have been studied as potential therapeutic targets as well as prognostic markers. NENs most commonly occur in the neuroendocrine system of the pancreas, gastrointestinal tract and lung, their grade being classified according to the percentage of Ki67 positive cells ranging from under 3% (G1), 3–20% (G2) to over 20% (G3) [3,4,5]. Chromogranin A (CgA) is a key biomarker in patients with NENs and is an indicator of development/progression due to its widespread expression in neuroendocrine tissues [1]. Despite its universal use, its sensitivity and specificity in the context of NEN are still under debate, as non-oncologic conditions may affect CgA, limiting its value [6].

On this note, CgA has been shown in patients with Hepatocellular carcinoma (HCC) with low diagnostic discrimination between cirrhosis and cancer [7], correlated with the degree of enterochromaffin-like cell proliferation in atrophic body gastritis [8] and has been reviewed for inflammatory bowel diseases [9], e.g., suggesting CgA may be implicated in inflammatory signaling in that context [10]. Hence, understanding the functional role of CgA may help in the future treatment of multiple cancers that affect secretory tissue.

Commonly released from many NEN as a secretory protein, full-length CgA may undergo proteolytic cleavage via prohormone convertases or plasmin, resulting in a multitude of bioactive fragments such as vasostatin, catestatin, and pancreastatin, each exerting distinct physiological effects affecting endocrine, cardiovascular, immune systems as well as glucose and calcium homeostasis [11]. Vasostatin/catestatin have been shown to induce calcium entry into neutrophils [12], which may directly induce Nlrp3 inflammasome activation [13].

Overall, neuroendocrine neoplasms range from the low-proliferating G1 neuroendocrine tumor to highly proliferative and poorly differentiated neuroendocrine carcinoma (NEC), with highly variable prognosis, survival and disease outcomes [14]. Treatment options comprise curative surgery, locally ablative therapies and systemic therapies using somatostatin analogues like octreotide and lanreotide, mainly affecting hormone secretion, the mTOR inhibitor everolimus, chemotherapy such as cis-platinum, 5-fluorouracil (5-FU) and temozolomide (TEM), and peptide receptor radionuclide therapy (PRRT) [15].

Owing to its high grade of vascularization [16] that comes alongside a well-adjusted tumor microenvironment [17], recent research has emphasized tumor–immune cell interaction, looking into T-cell [18,19,20] and macrophage interactions [21]. Our previous studies have shown initial evidence of inflammatory mediating miRNA including miR-29 that correlate with CgA serum levels [22], and miR-223 that was shown to be decreased in a cohort of patients including 60% G1 and 33% G2 [23], indicating that the anti-inflammatory miR-223 is altered in earlier stages of disease, and hence may be an indicator of immune cell activation. The specific function of miR-223 has been reviewed extensively by us [24] and others [25,26]. miR-223 has been linked to proliferation by mediating cyclin E activity [27], IGF-1R [28] or interaction with E2F1 [29].

In benefiting proliferation but also apoptosis, miR-223 has been shown to have pro and anti-oncogenic functions that have been reviewed extensively, e.g., HCC development has been shown to be ameliorated by miR-223, which was shown to be essential to hepatocyte maintenance via Taz/Hippo signaling [30], suggesting its role as therapeutic target and biomarker greatly depends on disease context, ongoing therapy and immune cell activation [31]. The strongest evidence for the role of miR-223 lies in its modulatory function of the nucleotide-binding oligomerization domain-like receptor (NLR) family pyrin domain-containing 3 (Nlrp3) and affecting inflammatory disease progression [32,33], especially stemming from [34,35], and affecting neutrophils but also monocytes/macrophages in vivo [36].

The aforementioned Nlrp3 inflammasome detects a range of endo- or exogenous danger signals that include pathogens and damage-associated triggers [37]. Assembly of the complex includes the activation of caspase 1, that cleaves pro-forms of IL-1β/IL-18, rendering them active as they are released by gasdermin-formed pores, which also may lead to a form of inflammatory cell death termed pyroptosis [38]. To limit excessive release of proinflammatory cytokines the constitutive activation of Nlrp3 via TLRs is controlled by autophagy via SQSTM1/p62 [39], but also the para/autocrine secretion of IL-10 [40]. By regulating immune cell activation, cellular proliferation and cell death in various organs [41] the inflammasome has been shown to mediate antitumoral immunity [42] but also cancer-promoting function [43]. Short-term activity may be conferring to antitumoral activity, long-term activation may benefit various forms of cell death possibly leading towards cancer [44]. miR-223 is linked to inflammasome regulation in neutrophils and was found to be altered in NEN [23], and our previous work identified increased serum IL-10 as indicator of stable/remissive disease, we aimed to identify possible mechanisms linking miR-223 to immune cell activation in NEN [45]. Therefore, we sought to identify a possible role of miR-223 in the tissue of liver metastatic NEN, evaluated the corresponding immune cell profiles, and additionally performed translational experiments to identify possible interactions between tumor, miR-223, Nlrp3, and immune cell response.

## 2. Materials and Methods

### 2.1. Patient Cohort

From January to September 2023, 10 patients diagnosed with neuroendocrine neoplasms were enrolled in our study at the tertiary center for neuroendocrine tumors at the Charité University Hospital. Prior to sample collection, patients informed written consent was obtained. The local ethics committee approved the study (EA1/229/17). Blood samples from patients were taken at or before the beginning of a treatment cycle. For retrospective histological analysis of pancreatic NEN and corresponding metastasis in liver, we collected samples (n = 5) between 2006 and 2019 approved under the local ethics committee und the registration numbers (EA1/229/17). Primary tumors were G2, Ki67 index between 2–10%, 3 males and median age of 65/average of 62.6 years.

### 2.2. Blood Flow Cytometry Preparation

Anti-coagulated (EDTA) whole blood was obtained and processed immediately. In total, 200 μL of whole blood was incubated with NIR fixable viability dye (1:5000, 423106 Biolegend, San Diego, CA, USA) and all fluorochrome-conjugated antibodies in blocking buffer (1:200) PBS 2% BSA (+2% normal mouse/human/rabbit/rat serum) mentioned in Table 1 for 20 min at room temperature. The remainder of blood was centrifuged at 1800× *g*/4 °C 10 min and frozen at −70 °C for the collection of serum/measurement of miRNA. Lysis of red blood cells was performed using standard Ammoniumchloride (20 mM)-Tris (50 mM)-buffer (pH 7.4) for 10 min. Intracellular staining was performed using True-Nuclear (Biolegend, San Diego, CA, USA) kit according to manufacturer’s instructions; all antibodies indicated in gray below were additionally stained intracellularly at a 1:100 dilution. After the final wash, cells were resuspended in a 200 μL staining buffer for analysis. Samples were analyzed on a standard issue Cytek (Fremont, CA, USA) Aurora Cytometer in a 3-laser setup using Spectroflo^®^ v3.0.3. Instrument QC was performed daily according to the manufacturer’s instructions. Samples were acquired at a low to medium speed. Data analysis was performed with FlowJo v10.6.2 (Ashland, OR, USA).

### 2.3. Fam-Flica Caspase 1 Activity Assay/CgA

Preparations of cells for flow cytometry applications were performed according to the manufacturers protocol (#98 ImmunoChemistry, Davis, CA, USA). In brief, cells were incubated with prediluted Flica-Reagent at 37 °C for 1h in their original culture medium. After removal, cells were incubated with apoptosis wash buffer twice at 37 °C for 15 min and counterstained with Hoechst33342 or Propidum Iodide (ImmunoChemistry, Davis, CA, USA ) and fixed according to manufacturer’s protocol. For staining of CgA, the samples were permeabilized (as above) and incubated with anti-CgA (NBP2-34674 Novus Biologicals, Minneapolis, MN, USA) in PBS 2% BSA for 20 min at 4 °C, washed and consecutively incubated for 30 min with anti-mouse Alexa Fluor-488 (A-11001/Thermofisher, Waltham, MA, USA), washed and fixed with 1% PFA for 10 min. Samples were immediately measured at BD (Franklin Lakes, NJ, USA) FACSCanto II Flow Cytometer equipped with (405/488/633 nm laser and 450/50 585/42 and 660/20 nm filtersets).

### 2.4. Microscopy and Microdissection

For staining of OCT (Optimal Cutting Temperature compound) cryosections samples were air dried for 5 min at RT, incubated with methanol at −20 °C for 10 min and blocked with 2% goat serum for 20 min. In the following slides, they were incubated with FAM-Flica reagent for 1h at 37 °C in a humid chamber, washed twice with apoptosis wash buffer at 37 °C for 15 min, consecutively stained with primary/secondary antibodies, and counterstained with Hoechst 33342 before imaging.

### 2.5. Immunofluorescence Microscopy

Cells were fixed with methanol at −20 °C, blocked with 2% goat serum and incubated with primary antibodies Synaptophysin ab178412 (1:400 Abcam, Cambridge, UK), PCNA antibody ab18197 (1:400 Abcam), Ki67 ab16667 (1:400 Abcam), CgB 18065364 (1:200 Novus Biologicals, Minneapolis, MN, USA) and CgA NBP2-34674 (1:200 Novus Biologicals, Minneapolis, MN, USA) in TBS 2% BSA overnight at 4 °C and secondary antibodies Alexa-488/Alexa-546/Alexa-647 (1:500 A-11001/A-11003/A-21447 Thermofisher, Waltham, MA, USA) for 3 h at room temperature followed by a Hoechst counterstain and mounting in aqueous medium. Images were taken at Zeiss observer 7 (Carl Zeiss AG, Jena, Germany) with Colibri Illumination and standard filter sets for DAPI/GFP/CY3/CY5.

### 2.6. RoboPALM/Microdissection

Samples were cut to 15 µm and a Cresyl violet/Hematoxylin quick staining. Retrieval of tissue from cryosections was performed using Zeiss RoboPALM/PALM microbeam at 200× magnification. Tissue was obtained from tumor and non-tumor areas at least 200 µm distant from the tumor border and was collected in AdhesiveCap 500 (415190-9201-000 Carl Zeiss AG, Jena, Germany). For quantitative analysis of miR expression, the cut area was used to normalize the results from the qPCR.

### 2.7. Cell Culture and Transfection

Pancreatic BON cells and IMAC inflammasome reporter cells were cultured in 1:1 DMEM: F12 + 10% FBS + 1% Penicillin/Streptomycin 37 °C/5% CO_2_. All experimental procedures/transfections were carried out at a target confluency of 50–70% if not mentioned otherwise. Transfection/miRNA knockdown was performed using the Attractene Transfection Reagent (301005 Quiagen, Hilden, Germany) and the si_miR-223 FlexiTube siRNA Premix (1027420 Quiagen, Hilden, Germany) according to the manufacturer’s recommendation in a reverse setup, performing the transfection during passage of cells. To avoid prolonged exposure to the reagent and to avoid transmission of complexes during tissue culture SN transfer medium was exchanged 12 h after transfection. If not mentioned otherwise, readouts/SN collections were performed an additional 36 h later (48 h after initial transfection). For the proliferation/scratch assay, a confluent culture was scratched with a p200 pipette tip, images were taken at *t* = 0 h and *t* = 48 h, the distances between edges of cells were measured to quantify migration/proliferation in either condition. The actin of BON was stained using Alexa Fluor™ 350 Phalloidin (A22281/Thermofisher, Waltham, MA, USA) according to manufacturer’s instructions. In IMAC culture, reporter cells were treated for 2 h with 5 µg/ml Nigericin (J61349.MA Thermofisher, Waltham, MA, USA) to induce activation of the Nlrp3 inflammasome. The functional application of the Nlrp3 inflammasome reporter cell line has been described previously [46].

### 2.8. BrdU

Evaluation of cell proliferation using 5-bromo-2′-deoxyuridine to quantify was performed using BrdU Staining Kit for Flow Cytometry (Invitrogen/Thermofisher, Waltham, MA, USA) according to manufacturer’s instructions. BrdU was added to the cell culture after removal of the transfection complexes, and then cultured for an additional 36 h.

### 2.9. Primary Cell Isolation

For monocyte isolation, whole EDTA blood from healthy donors was mixed 1:1 with PBS and layered on top of a Percoll 1.131g/mol (17089101 Cytiva, Marlborough, Massachusetts, USA) density gradient medium, and centrifuged at 600× *g*/20 min at room temperature without break. The layer containing mononuclear cells was retrieved, washed and transferred to cell culture plates. After 3 h, non-adherent cells were removed and remaining adherent cells cultured in DMEM 10% FBS + 1% Penicillin/Streptomycin at 37 °C/5% CO_2_ + MCSF (50 ng/mL, 300-25 Peprotech, Waltham, MA, USA) for 7 days. For neutrophil isolation heparinized blood was mixed with 5% Dextran (Sigma-Aldrich, St. Louis, MO, USA) and 5 ml PBS w/o Ca^2+^ and Mg^2+^ for 30 min to allow leukocyte sedimentation. The upper phase was collected and slowly placed on top of 3.3 mL Ficoll-Paque Plus (GE Healthcare, Chicago, IL, USA), to further separate polymorphonuclear from mononuclear cells and erythrocytes. After centrifugation for 20 min at 600× *g* at room temperature without brake, cells were washed with HBSS and cultured in DMEM + 10% FBS + 1% Penicillin/Streptomycin at 37 °C/5% CO_2_. Cells were pre-treated with medium or 30% cell culture SN (in medium) for 10 h and consecutively treated with 500 ng/ml *E. coli* O128:B12 Lipopolysaccharides (Sigma-Aldrich, St. Louis, MO, USA) for 6 h or Zymosan A (*S. cerevisiae*) BioParticles (Thermofisher, Waltham, MA, USA) for 30 min.

### 2.10. qPCR miRNA and Regular SYBR Green

Cell pellets/microdissected samples were resuspended in 500 µL Trizol reagent and mixed with 400 µL chloroform, for miRNA isolations the samples were spiked with SV40 spike in miRNA mimic (miScript miRNA mimic, 219600, Quiagen, Hilden, Germany) and incubated at RT for 10 min. After centrifugation at 13,000× *g*, 15 min, 4 °C clear supernatant was mixed with equal parts of isopropanol and 1.5 µL glycogen and incubated o/n at −20 °C. Following centrifugation at 13,000× *g*, 30 min, 4 °C the pellet was eluded using 80% Ethanol and resuspended in RNAse free water. For miRNA preparation samples were transcribed using the QuantiTect Reverse Transcription Kit (205311, Quiagen, Hilden, Germany) and QuantiTect Primer Assay (249900, Quiagen, Hilden, Germany) for miR-155; miR-193; miR-223 and SV40 combined with QuantiTect SYBR Green PCR Kit (204145, Quiagen, Hilden, Germany) according to the manufacturer’s instructions. Other samples were transcribed using the iScript cDNA synthesis kit (1708890, Bio-Rad, Hercules, CA, USA) according to the manufacturer’s instructions. qPCRs were performed using PowerUp SYBR Green Master Mix (A25742 Thermofisher, Waltham, MA, USA). All qPCR reactions were run on a QuantStudio3 machine (Thermofisher, Waltham, MA, USA). For comparison of BON cell culture, results were normalized to the control condition using the expression of a control gene (GAPDH) results are expressed as relative quantity (RQ) using 2-deltaCT (Table 2). For the evaluation of miR expression in samples isolated from cryosections, intrasample normalization was performed using SV40, and the RQ was calculated by normalizing extra- towards intratumoral tissue and corrected for in the microdissected area.

### 2.11. Western Blotting

Western Blotting was performed according to standard protocols. In total, 50 µg protein in RIPA buffer were loaded on AnyKD Mini-PROTEAN^®^ TGX™ Precast Gels (4569033), transferred using Trans-Blot Turbo Transfer System (1704150) nitrocellulose membranes (1704158) blocked with EveryBlot Blocking Buffer (12010020) for 30 minutes. CgA Antibody (1:400) o/n and secondary StarBright Blue 520/700 (12005866/12004158) (1:4000)/Rhodamine anti GAPDH (12004168/1:5000) for 2h. Pictures were taken on a ChemiDoc MP system (12003154) and band sizes evaluated using the corresponding image lab software (v.6.0.1; all Bio-Rad, Hercules, CA, USA).

### 2.12. Sequencing and Correlation of Inflammatory/NEN Marker Expression

The data for analysis of gene expression in healthy liver (n = 3), healthy pancreas (n = 6), G1 (n = 14), G2 (n = 24) and G3 (n = 19) NEN were pulled from the publicly available datasets published by Otto et al. in 2023 [47]. Statistical analysis was performed using Tukey’s multiple comparison test, simple linear regression for correlations and Pearson r models with a two-tailed P and 95% confidence interval.

### 2.13. Statistical Analysis

Analyses were performed with Graph Pad (version 9.5.1; GraphPad, GraphPad Software Inc., Boston, MA, USA). Evaluation of statistical significance was determined as follows: two groups were compared using unpaired t-test; three or more groups using ordinary one-way ANOVA followed by Tukey’s multiple comparison test. Correlation analyses were performed using Pearson correlation coefficient. Linear regression, 95% confidence intervals, and Area Under Curve were calculated using the corresponding functions in the software. The significance level was set at α = 5% (* *p* < 0.05; ** *p* < 0.01; *** *p* < 0.001; **** *p* < 0.0001) for all comparisons. Unless otherwise stated, data are expressed as mean +/− SEM or as an absolute number or percentage for categorical variables.

## 3. Results

### 3.1. Serum miR-223 Expression Correlates with Circulating Neutrophil Markers in NEN

**Figure 1:**
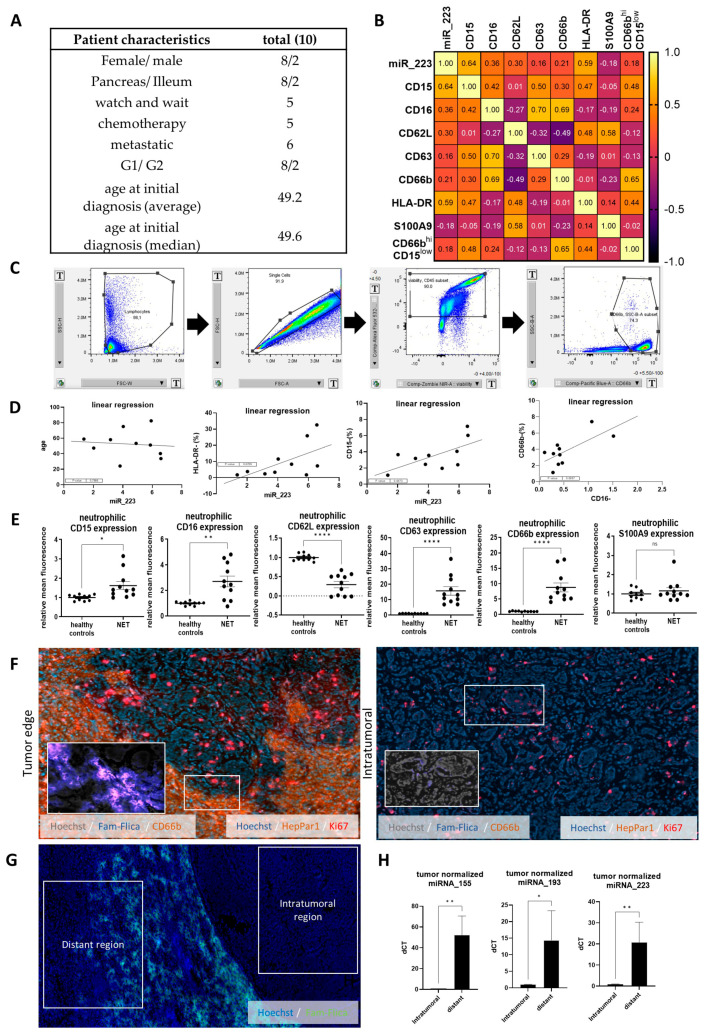
miR-223 correlates with neutrophilic markers in vivo in a small cohort of patients with NEN. (**A**) Characteristics of the heterogenous group of patients (n = 10). (**B**) Pearson correlation matrix including miR-223 serum levels and marker expression in the neutrophil population. (**C**) Gating strategy for neutrophils from patient samples. (**D**) Linear regression of miR-223 and selected markers (data from (**B**)). (**E**) Control cohort normalized mean fluorescence intensity of markers in the neutrophilic population, neutrophil marker expression in healthy controls and NEN patients (n = 11/10). (**F**) Neuroendocrine metastases in the liver were identified by the absence of HepPar1 staining, neutrophils were identified by CD66b and proliferation was determined by Ki67 staining. Small section cutouts show Fam-Flica (Cl. caspase-1 staining)/CD66b staining in relation to HepPar1/Ki67 staining (**G**) Fam-Flica (Cl. caspase-1 staining) staining in the liver of patients with metastatic NEN. Intratumoral and tumor-distant regions were identified using HepPar1 staining (as in (**F**)). (**H**) Tumor tissue and tumor-free tissue from tumor distant regions were isolated via microdissection. Microdissected samples from the tumor and distant region (exemplary in (**G**) were normalized towards the resected area and spike-in miRNA. Plotted are relative quantities towards each individual intratumoral sample (n = 5) (* *p* < 0.05, ** *p* < 0.01, **** *p* < 0.0001, paired *t*-test).

To evaluate the role of microRNA in the context of primary and metastatic NEN we initially performed a pilot study including a detailed evaluation of circulating neutrophils (Figure 1A). Our cohort consisted of 10 patients, including 8 pancreatic NEN, half of them with metastasis including 2 with G2 status, 2 with ileal NEN, 6 with metastatic NEN, 5 undergoing chemotherapy, including 1 patient with illeal NEN and 4 with pancreatic NEN, 2 of each with G1 and G2 status. Those parameters were correlated with the frequency of blood immune cell populations. However, we did not observe significant correlation between patient characteristics and blood immune cell composition. Therefore, we additionally performed a Spearman and linear regression analysis to additionally include miR-223 serum values (Figure 1B). As expected, we found a significant correlation between neutrophilic CD16 and CD63 (*p* = 0.025, confidence interval 0.123 to 0.922) as well as CD66b (*p* = 0.026, CI 0.116 to 0.921), we also found a significant inverse correlation between CD15 and miR-223 expression (*p* = 0.047, CI 0.0136 to 0.904) (Figure 1D). To compare neutrophilic marker expression between healthy controls and the NEN cohort we compared the mean fluorescence intensity of each marker in the neutrophil population, following a normalization to the control cohort. We found significant increase in CD15 (relative fluorescence 1 to 1.6, *p* = 0.0125, CI 0.1501 to 1.094), CD16 (1 to 2.7, *p* = 0.0011, CI 0.7793 to 2.644) and striking increases in CD63 (1 to 15.7, *p* < 0.0001, CI 8.593 to 20.87), CD66b (1 to 8.7, *p* < 0.0001, CI 4.588 to 11.00) while CD62L was decreased (1 to 0.29, *p* < 0.0001, CI −0.8948 to −0.5168) and S100A9 remained unaltered (1 to 1.2, *p* = 0.416, CI −0.2411 to 0.5653). Altogether, this suggests an increased activity of neutrophils in NEN (Figure 1E).

As decreased miR-223 has been previously shown in the serum of mixed pancreatic/ileal G1-G3 mostly metastatic NEN, we here looked into local distribution of various miRs in NEN metastasis and surrounding tissue. To allow discrimination and evaluate the role in only progressed liver metastatic NEN we accordingly performed immunofluorescence staining on fresh–frozen tumor samples from liver metastases. NEN metastases in the liver were clearly distinguished from liver tissue by the absence of the hepatocyte-specific marker HepPar1. Quite strikingly, caspase-1 activity was vastly excluded from the tumor regions, and was most prominently found alongside CD66b positive neutrophilic cells at the tumoral intersection (Figure 1F). Accordingly, we performed microdissections of intratumoral and tumor-distant regions for further evaluation. Quantitative analysis showed the near absence of miR-223 (1 to 20.7, *p* = 0.0039), miR-155, as well as miR-193 in the metastasis (Figure 1H), alongside a drastic increase in caspase-1 activity in the surrounding tissue (Figure 1G). In summary, we report an extratumoral increase in miRNA that overlaps with neutrophils and caspase-1 activity, which are mostly excluded from the metastatic tissue.

### 3.2. Knockdown of miR-223 Promotes Expression of NEN Differentiation Markers

**Figure 2:**
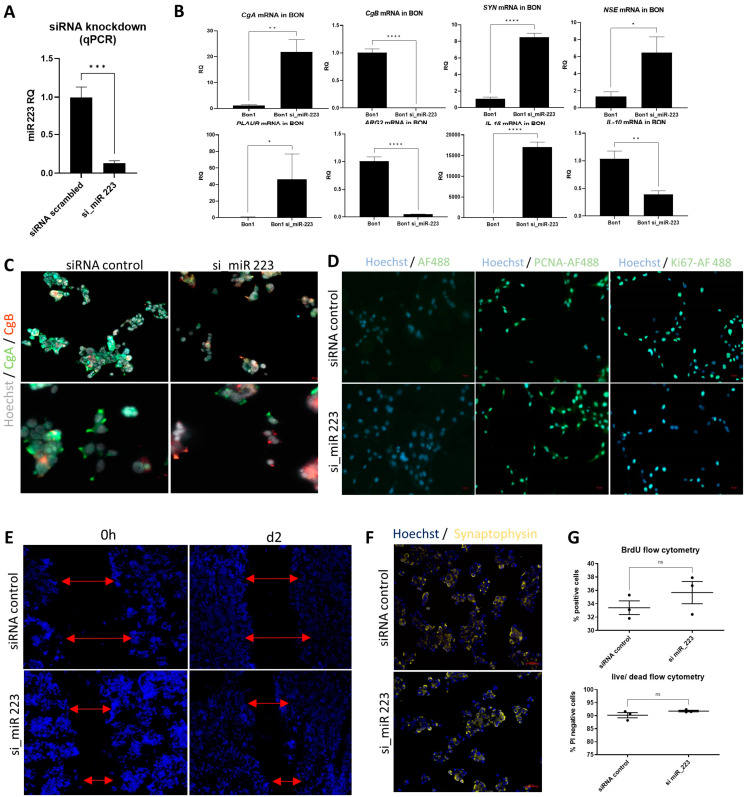
miR-223 knockdown in vitro on BON alters inflammatory gene expression. (**A**) In vitro knockdown of miR-223 in BON cells using a reverse transfection results in decreased miR-223 transcripts compared to scrambled-control transfected cells after 48 h. (**B**) In vitro knockdown of miR-223 strongly affects gene expression of neuroendocrine and inflammatory markers, displayed are relative quantities normalized towards gene expression in scrambled-control transfected cells (n = 3–4) (* *p* < 0.05; ** *p* < 0.01; *** *p* < 0.001; **** *p* < 0.0001, unpaired *t*-test). (**C**) Immunofluorescence staining of CgA/CgB in BON following the knockdown of miR-223 (200×/400× magnification, bar indicates 20 µm). (**D**) Immunofluorescence staining of BON including staining control (**left**), PCNA (**middle**), and Ki67 (**right**) (200× magnification). (**E**) Following 48 h transfection, a confluent culture was scratched and evaluated after additional 48 h of culture. Red arrows indicate the distance between nearest cells at *t* = 0 h. (50× magnification). (**F**) Immunofluorescence staining of Synaptophysin in BON following the knockdown of miR-223 (200× magnification). (**G**) Flow cytometry of BON, stained either with BrdU for cellular proliferation and propidium iodide, negative cells indicate survival (unpaired *t*-test, not significant *p* > 0.05, n = 3).

To evaluate the role of miR-223 on aspects of tumor biology in detail, we performed in vitro siRNA-induced knockdown of miR-223 in the established NEN cell line BON. We therefore either used si_miR-223 (si_miR-223 BON)-transfected cells or scrambled miRNA-transfected (BON) as control. miR-223 levels were decreased to about 10% of their baseline level (*p* = 0.0007) after 48 h (Figure 2A). All controls mentioned from here on were similarly transfected with scrambled siRNA. Consecutively, we assessed the gene expression of common neuroendocrine differentiation markers, showing a distinct upregulation after 48 h for *CGA* (RQ 21.7/*p* = 0.0051), *NSE* (RQ 6.4/*p* = 0.038) and Synaptophysin (*SYN*) (RQ 8.4/*p* < 0.0001), while *CGB* was significantly decreased (RQ 0.0028/*p* < 0.0001) (Figure 2B). The miR-223 knockdown also affected parameters such as *PLAUR*, *IL-10*, *IL-1β* and *Arginase2*. Immunofluorescence staining of BON cells showed a decrease in CgA, alongside an increase in CgB signal following miR-223 knockdown (Figure 2C), while synaptophysin expression was not visibly altered (Figure 2F). We evaluated the cellular proliferation, showing minor increases in the proliferation markers Ki67 (Figure 2D) and BrdU (Figure 2G), though not reaching statistical significance. Cell death was similarly not affected by the knockdown (Figure 2G). A scratch assay performed to evaluate migratory and proliferative properties also showed no significant difference following the knockdown of miR-223 over the time of 48 h, suggesting no effect with regards to proliferation/migration in the short time (Figure 2E). This indicates the short-term effect of the knockdown mostly affects neuroendocrine marker/inflammatory activation, but not the proliferation or migration of BON.

### 3.3. Alpha-2-Antiplasmin Reverts miR-223 Induced Phenotype in NEN Cells

**Figure 3:**
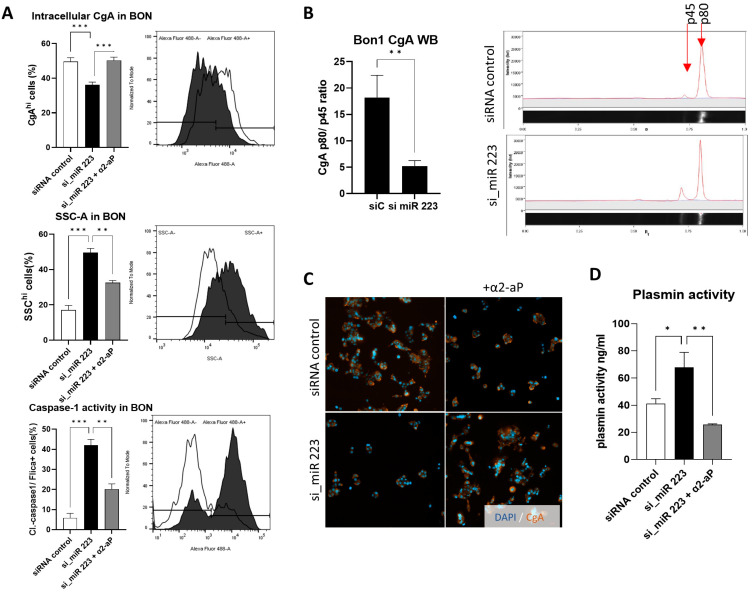
miR-223 knockdown induces CgA release and Nlrp3 inflammasome activation in BON cells. (**A**) Flow cytometry analysis of BON following the knockdown of miR-223 shows altered CgA, granularity and caspase-1 activity. Exemplary histograms, and quantification (n = 4) ordinary one-way ANOVA was performed, ** *p* < 0.01. (**B**) The ration of CgA protein subunits p80 and p45 (indicated on the right, graphical representation using abstracted lane fluorescence) are altered between control and knockdown. Bar graph indicates p80 to p45 ratio (n = 3, significance of ** *p* < 0.01 was calculated with an unpaired *t*-test). (**C**) Exemplary immunofluorescence of CgA in BON following miR-223 knockdown and α2-aP treatment (200× magnification). (**D**) Plasmin activity in BON samples to evaluate the inhibitory efficacy of α2-aP in ng/ml (n = 4, * *p* < 0.05; ** *p* < 0.01, *** *p* < 0.001, statistical analysis was performed using ordinary one-way ANOVA).

To further assess the characteristics quantitively, we performed a flow cytometric evaluation of BON cells, staining CgA as well as Caspase-1 activity intracellularly. The number of CgA^hi^BON was significantly decreased following the knockdown of miR-223 (49.6 to 36.2, *p* < 0.0001, Figure 3A). Additionally, we found a striking increase in cellular granularity (SSC), and cells with active caspase-1 (Flica^+^ 5.9 to 42%, *p* = 0.0002).

Performing Western Blots of BON lysates, we found an increase in cleaved p45 of CgA. The ratio between p45 and p80 was found to significantly increase in favor of the cleaved fraction from 18.2 to 5.2 (*p* = 0.0066) in si_miR-223-treated BON (Figure 3B). In order to address the proteolytic cleavage of CgA, we used alpha-2-antiplasmin (α2-aP) to inhibit plasmin-dependent processing of CgA. Inhibition efficiency was determined by measuring plasmin activity in BON, which showed limited activity in the presence of α2-aP (41.3 to 67.9 to 25.7 ng/ml, * *p* < 0.05/** *p* < 0.01), while the knockdown strikingly increased enzymatic activity (Figure 3D). Again, flow cytometry showed that α2-aP fully ameliorates the effect of miR-223 knockdown with regards to the number of CgA^hi^BON (36.2 to 50.3%, *p* < 0.0001) while cellular granularity (SSC^hi^ 49.5 to 32.5%; *p* = 0.0037) and caspase-1^+^ (Flica^+^ 42 to 20.2%; *p* = 0.0026) activity were significantly reduced. Complementary immunofluorescence staining of CgA provided visual indications of the restored CgA expression in the presence of α2-aP (Figure 3C).

### 3.4. Neutrophils Are Activated Following Stimulation with BON Supernatant (SN)

**Figure 4:**
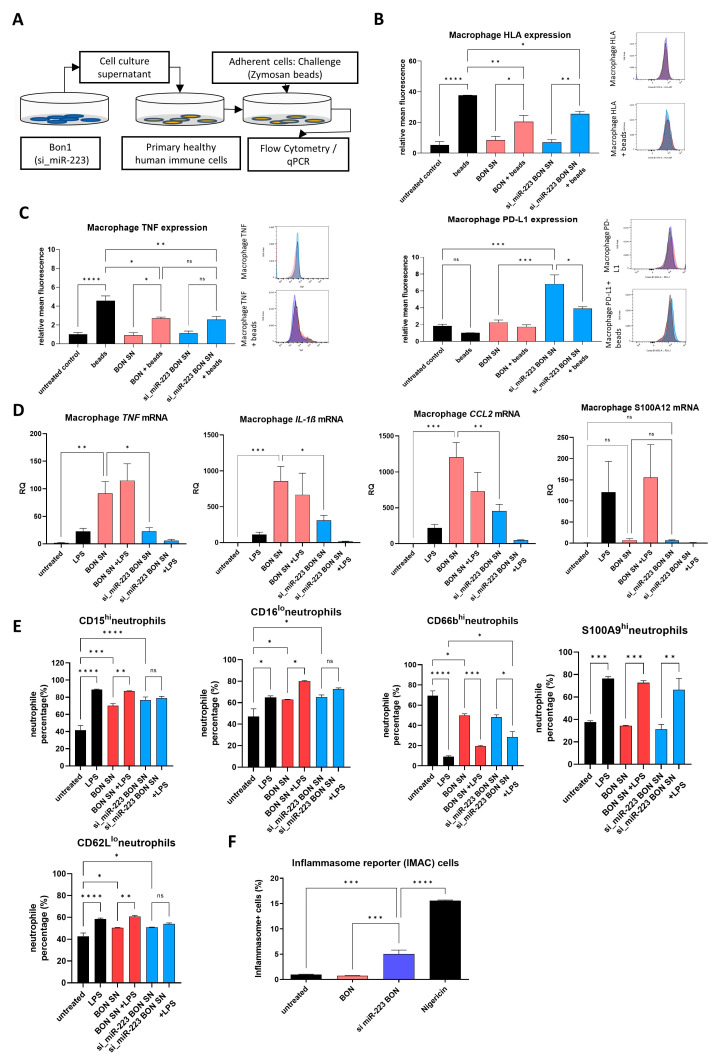
(**A**) The SN of (si_miR-223 transfected (blue) or control transfected (red) or not SN treated (black)) BON cells was used to treat primary human immune cell populations for 10 h in vitro and consequently challenged with zymosan beads or LPS to evaluate subsequent immune responses. Flow cytometry of CD14^+^/CD11b^+^ macrophages, (**B**) PD-L1, (**C**) TNF and HLA expression and exemplary histograms (n = 3, * *p* < 0.05; ** *p* < 0.01, comparison calculated using ordinary one-way ANOVA), (**D**) qRCR of macrophages treated with LPS, shown are relative quantities, normalized to untreated controls. (n = 4, * *p* < 0.05; ** *p* < 0.01, *** *p* < 0.001, comparison calculated using ordinary one-way ANOVA), (**E**) CD66b+/SSChi neutrophilic cells flow cytometry for corresponding marker expression with and without LPS challenge. (n = 3), (**F**) IMAC reporter cells cultured in the presence of BON SN and challenged with nigericin, active Nlrp3 inflammasome formation is indicated as percentage of positive cells measured in flow cytometry. (n = 3) The presented histograms are representative of the experimental data. (n = 3; * *p* < 0.05; ** *p* < 0.01, *** *p* < 0.001, **** *p* < 0.0001, comparison calculated using ordinary one-way ANOVA).

As the knockdown of miR-223 led to an activated Nlrp3 inflammasome, we consequentially questioned whether altered miR-223 may potentially affect immune cell responses. To further explore this question, we collected the supernatant (SN) of control/si-miR-223-transfected cells. This SN was consequentially used to treat primary human blood neutrophils and macrophages from healthy donors. Macrophages were further treated with zymosan-coated beads to determine their response to phagocytosed particles. The impact on neutrophil and macrophage activation was determined by flow cytometry. (Figure 4A). Notably, the number of macrophages was determined by CD11b^+^/CD14^+^, and was generally above 94%.

The already low expression of PD-L1 on untreated macrophages was further decreased upon challenge with zymosan beads. Strikingly, si_miR-223 SN significantly induced relative mean fluorescence intensity in PD-L1 compared to untreated controls, while control transfected BON SN did not (1.9 or 2.3 to 6.8, *p* = 0.0001/*p* = 0.0003) (Figure 4B). Accordingly, control SN did not affect baseline expression of TNF or HLA. Following stimulation with zymosan, macrophages treated with either BON SN failed to reach similar expression levels compared to the controls for TNF (4.6 to 2.7 or 2.56, *p* = 0.0126/*p* = 0.0074) and HLA (37.7 to 20.4 or 25.5, *p* = 0.0026/*p* = 0.032) (Figure 4C). Remarkably, gene expression of inflammatory markers in response to LPS was mostly increased in BON SN-treated cells (Figure 4D). si_miR-223 SN reduced gene expression in TNF (RQ 91.6 to 22.7, *p* = 0.0125), IL-1β (RQ 858.7 to 310.1, *p* = 0.0168) and CCL2 (RQ 1204.8 to 456, *p* = 0.0037) while either the SN-treated condition rendered the cells less responsive towards LPS compared to the control condition aside from S100A12, which failed to show any increase in the si_miR-223 SN + LPS condition (RQ 120.9 to 155.5 to 1) (Figure 4D).

Neutrophils were identified by SSC-A^hi^/CD66b^hi^ expression; following the isolation the ratio was generally over 90% of life cells. We found less CD15^hi^ and CD16^lo^ cells in either BON SN-treated condition, there was no visible difference after 6h of LPS between all conditions (CD15^hi^ 89.1 to 87.3 to 79%) (Figure 4E). S100A9 was virtually unaltered between either condition and similarly increased upon challenge. While CD62L^lo^ cells were significantly increased upon LPS in control (42.8 to 58.5%, *p* < 0.0001) and BON SN (50.4 to 60.8% *p* = 0.0019), si_miR-223 SN-treated cells failed to show significant increases upon challenge (50.8 to 54%, *p* = 0.58). BON SN and si_miR-223 SN increased the amount of CD62L^lo^ cells significantly, while the number of CD66b^hi^ neutrophils decreased (69.4 to 49.9 or 48%, *p* = 0.0107/*p* = 0.0053). Upon LPS challenge, the amount was reduced in the control; si_miR-223 SN-treated cells showed significantly less degranulation of CD66b (9.1 to 28.3%, *p* = 0.0119), while BON SN did not.

To validate our hypothesis that miR-223 regulation in BON indirectly affects Nlrp3 inflammasome activation in immune cells, we transferred cell culture SN to the Nlrp3 inflammasome reporter cell line IMAC, which forms ASC-Nlrp3 specks that can be evaluated using flow cytometry. We found that BON SN failed to induce Nlrp3 inflammasome activation, si_miR-223 SN eventually increased the speck formation in those cells significantly (1 to 5.1%, *p* = 0.0004), reaching about a third of the full inflammatory potential of the reporter cells treated with the specific inflammasome activator Nigericin (5.1 to 15.6%, *p* < 0.0001) (Figure 4F). This suggests the SN of si_miR-223 BON efficiently induces Nlrp3 inflammasome activation. We altogether find that si_miR-223 BON affects inflammation-related activation dependent on Nlrp3, while regulatory functions including PD-L1 are similarly induced in macrophage culture.

### 3.5. Co-Culture of miR-223 Knockdown in BON Induces Increased Tumor Cell Clearance by Neutrophils

**Figure 5:**
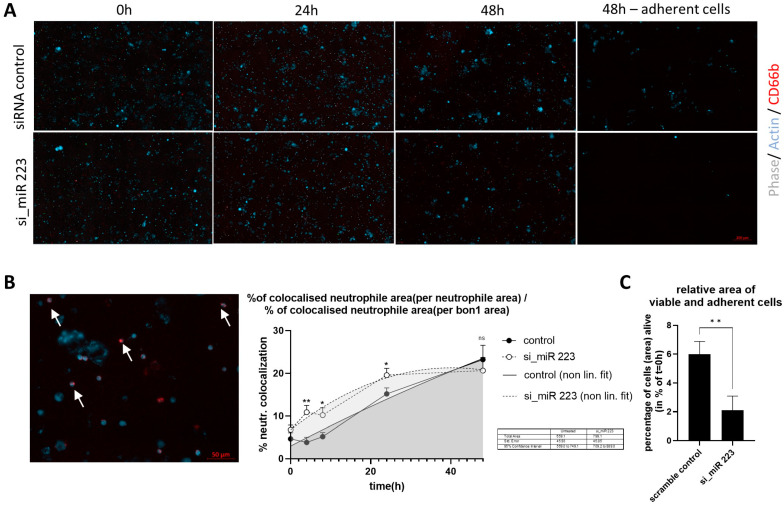
(**A**) BON cells were transfected for 48 h and labeled with an actin binding Alexa Fluor350 Phalloidin conjugate, the medium was replaced by a medium containing fluorescence-labeled (CD66b) neutrophils in a 1:3 ratio and cultured for additional 48 h. Pictures were taken after 2, 4, 24 and 48 h. The final picture was taken after removal of tissue culture SN containing cellular debris/ remaining immune cells. (**B**) Exemplary image showing overlap between phase contrast (white), actin in BON (blue) and CD66b^+^ (red) neutrophils. Total overlap was calculated over the course of 48 h, area under the curve was calculated using indicated timepoints (0, 4, 8, 24 and 48 h) (**C**) Percentage of actin positive area at *t* = 48 h compared to *t* = 0 h following the removal of SN. (n = 4, * *p* < 0.05, ** *p* < 0.01 was calculated using an unpaired *t*-test).

As previously established, we found that the knockdown of miR-223 in BON via SN transfer increased the inflammatory potential of neutrophils. We hence questioned if this had any effect on the clearance of “tumor” cells in an in vitro setup. Following 48 h of transfection SN and staining of actin in BON, we added primary neutrophils to the culture. Comparison of control/transfected cells throughout the experiment did initially not show strong differences (Figure 5A). To evaluate the number of live cells after the incubation time we carefully removed tissue culture supernatant, hence removing debris and dead cells. Calculation of total surface area covered compared to *t* = 0 h showed a significantly higher amount (6 to 2.1%, *p* = 0.009) of remaining live BON cells in the control transfected group (Figure 5C). In order to assess the interaction between immune and BON cells, we calculated the overlap between actin (BON cells) and CD66b signal (neutrophils) over time (Figure 5B). The area of colocalized neutrophils was then normalized to the total neutrophil and BON cell area. We found a significant increased neutrophil-BON cell interaction at 4 h (3.8 to 10.8%, *p* = 0.0037), 8 h (5.1 to 10.2%, *p* = 0.011), and 24 h (15.2 to 19.6%, *p* = 0.023), which declined at 48 h, (total AUC 569.0 to 749.1; 709.2 to 889) in the si_miR-223-transfected cells, indicating a longer interaction with neutrophils compared to the control/transfected condition.

### 3.6. Sequencing Data of G1-G3 NEN Indicate Correlations Between Inflammatory and Functional Genes

**Figure 6:**
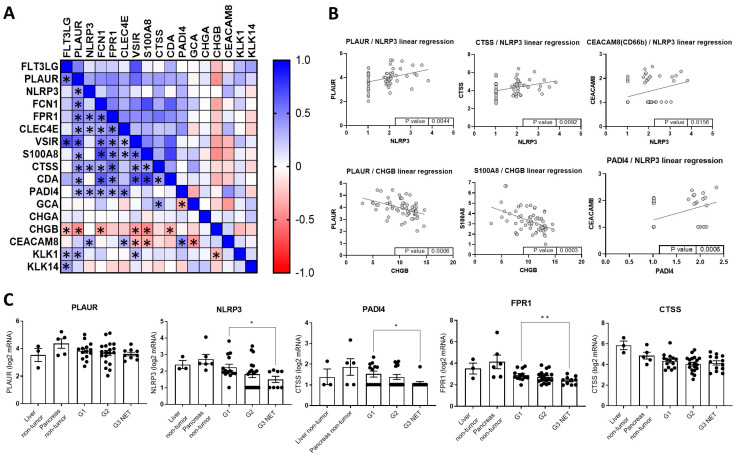
Evaluation of gene expression in a cohort of G1-G3 NEN derived from published sequencing data. (**A**) Asterisks indicate significant correlations between two genes, blue indicates positive, red negative respective influence. * *p* < 0.05. (**B**) Linear regression between two indicated genes, abstracted data from (**A**), *p* value indicates significant slope variation from zero. (**C**) Gene expression of indicated genes in G1-G3 NEN and control cohorts. (* *p* < 0.05; ** *p* < 0.01, comparison calculated using mixed effects analysis and Tukey’s multiple comparison test).

To verify the relevance of Nlrp3, CgA and Plasmin we looked into previously published sequencing data on G1-G3 NEN. Pearson correlation identified *PLAUR* as target gene that tallied well with many of the inflammatory-associated targets, including *NLPR3*, *S100A8*, Cathepsin S, MHC-controlling *PAD4* but also Chromogranin B (Figure 6A). *PAD4* unsurprisingly corresponded well with *NLRP3* (*p* = 0.0006), which showed a strong correlation with *CEACAM8* (CD66b) (*p* = 0.0156). *NLRP3* correlated with *PLAUR* (*p* = 0.0044), which in turn was inversely aligned with *CHGB* (p=0.0006). Evaluation of differential gene expression between G1 and G3 revealed a trend towards decreased expression of *FPR1* (2.9 to 2.4, *p* = 0.009), *NLRP3* (2.2 to 1.5, *p* = 0.044) and *PAD4* (1.5 to 1.1, *p* = 0.041). Notably, we found several patients without gene expression of *NLRP3* and *PAD4*, their number increased between G1 and G2-to G3 NEN.

## 4. Discussion

As we previously found, miR-223 is regulated in patients with ongoing NEN disease [23]; we therefore addressed the functional role of miR in different NEN tumor and how it impacts the function of neutrophils through Nlrp3. Previous studies have shown that a high neutrophil to lymphocyte ratio in tumors correlates with a poor outcome [48]. Although this correlation was not observed in our small study, likely due to the small sample size, we conducted a more detailed analysis of neutrophil function. High miR-223 significantly correlated with decreased expression of the adhesion molecule CD15 (Lewis X), it is generally considered to correlate with neutrophilic activation [49]. Correlation between additional neutrophilic markers including the phagocytosis marker CD16, granulocyte activation marker CD66b and CD62L/L-selectin have not been shown in NEN to this date. Quite strikingly, comparing NEN to healthy control samples we find increased CD63 and CD66b and decreased CD62L expression, indicating a specific activation of neutrophilic markers in the context of NEN. A recent study found specifically decreased CD62L in correlation with breast cancer metastasis, indicating those neutrophils may be tumor promotive [50], and CD62L^lo^ neutrophils benefited metastatic spread in melanoma models [51], hence warranting further evaluation if a similar mechanism is in NEN as well. While the findings presented here suggest a link between miR-223 and neutrophilic activation, this explorative patient study using a small number of patients is limited to the presented correlations in the neutrophil population itself, but is not powered to find correlations with the course of disease. Future evaluation of neutrophilic subpopulations would require a more refined approach, e.g., discriminating between tumor stage, grade, metastatic development and treatment, all of which could affect the degree of exposure towards the immune system and consequently affect neutrophilic activation. This would possibly allow for a better understanding of the neutrophils function depending on disease progression, which remains impossible in the setting presented here.

To identify the spatial distribution of miRNA expression in liver metastasis, we used the hepatocyte-specific marker HepPar1 [52], which was completely excluded from the NEN-tumor metastasis. Neutrophils were found solely outside of the tumors, which colocalized with active-caspase-1, hence indicating Nlrp3 inflammasome activation and suggesting an ongoing proinflammatory activity. Immune cell populations, especially neutrophils, are a major source of miR-223 [36], which is the likely reason we found those signals to spatially overlap in our samples. Contrastingly, all investigated inflammatory miRs in the metastatic tissue appeared to be comparably low, suggesting that there is no significant trafficking towards the tumor. Accordingly, our results show that miR expression stems from the tumor microenvironment, which accommodates plenty of neutrophils.

We assume that the latter are the source of miR-223, downregulating proinflammatory activity and possibly benefiting tumor survival. According to the recent literature the function of miR-223 greatly depends on tumor–immune cell interactions in the tumor microenvironment, making its role generally hard to predict for overall outcome [53]. Increased miR-223 levels may be linked to regulatory mechanisms reducing inflammation, hence benefitting tumor survival in this context. The functional data for miR-223 in various cancers have shown ambiguous roles with regards to proliferation and apoptosis, but also affecting inflammation [31]; hence, knowing its localization and target is relevant to predicting its function.

BON did not show a correlation between decreased miR-223, proliferation or increased apoptosis in our cell culture model, even though it has been previously shown that miR-223 expression correlated with disease progression, i.e., decreased tumor cell proliferation and increased apoptosis [54]. Studies using miR-223-deficient animals indicated a worse phenotype compared to wild type controls, which suggest that miR-223 is essential to mediating inflammatory signaling [30]. Questioning if those low amounts of miR directly benefit proliferation of tumor cells directly, or indirectly limits immune cell activation we performed in vitro knockdown of miR-223 in BON, a well-established CgA-expressing NEN cell line [55]. Notably, we performed similar experiments with QGP-1/H727 cell lines, but found them to be mostly unresponsive towards miR changes, which we attribute to the lack of/decreased secretion of CgA, which would be a prerequisite for the effect described here.

Whilst proliferation/wound-healing capacity or cell death were not affected, we found striking regulation of *CGA*/*CGB* and inflammatory parameters including proinflammatory *IL-1β* and negative regulation of anti-inflammatory *IL-10*. Notably, *ARG2,* inflammatory *IL-1β*, and *IL-10* have previously been described in the pancreas and pancreatic neoplasia [56,57,58]. Quantification of CgA in BON showed a clear decrease upon knockdown of miR-223, which came alongside a decreased ratio of CgA fragments p80/p45 [11]. Previous research has already shown proteolytic cleavage of CgA by plasmin [59], which is readily available on the surface of secretory cells [60], but also the interaction with tissue plasminogen activator with CgA on those cells has been recently established [61]. Limiting plasmin activity with α2-aP reverted intracellular CgA levels, but also reduced cellular granularity and caspase-1 activity. While CgA/CgB have been shown to impact the biogenesis of granules [62,63], we are unsure of how limiting extracellular plasmin activity affects intracellular granularity, which may be a question warranting further attention. We hypothesize that autologous signaling following CgA proteolytic cleavage induces an inflammatory feedback loop inducing Nlrp3 and induces CgA gene expression due to decreased cytoplasmatic levels. We also transfected miR-223 into BON, which had no effect on CgA and other readouts. This suggests that the downstream effect is directly mediated by the Nlrp3 inflammasome, which is mostly inactive in untreated BON, hence it cannot be additionally limited by additional miR-223.

To further evaluate if lowering miR-223 in BON affected the inflammatory potential of immune cells, we performed tissue culture SN transfer to primary in vitro differentiated macrophages or neutrophils. As has been previously established the programmed death ligand 1 (PD-L1) essential to monocyte differentiation [64] and dependent on Nf-kB signaling [65], we found a decreased expression of TNF and HLA with and without challenge in either BON SN-treated cells, while the si_miR-223 SN specifically induced the immunosuppressive PD-L1. Looking into neutrophils, we found si_miR-223 SN to increase CD15 while decreasing CD62L/CD66b, hence inducing a more active phenotype. Following a challenge with LPS, the response was markedly reduced compared to neutrophils cultured without SN, which suggests that while BON supernatants activate neutrophils pre-emptively, it renders them less responsive towards proinflammatory stimuli. To evaluate the direct BON–neutrophil interaction we performed co-culture experiments including miR-223 knockdown cells. The data provided evidence of a higher affinity of neutrophils for si_miR-223 BON over the control-treated cells. Of note, the difference in colocalization was not detectable past 48h, indicating that the miR-223 knockdown affects the migration initially, which may be linked to decreased CD62L, but possibly not in the long term.

Of note, the targeting of miR-223 has been previously performed, e.g., LPS-induced inflammation or fibrotic NASH models were mitigated by transfecting miR-223 [66,67]. Previous research has hinted towards ongoing inflammation and (increased) circulating inflammatory cytokines as possible biomarkers of disease progression [45,68], as they are possibly tumor promotive, especially affecting angiogenesis or metastatic spread [69]. While we approached altered miR-223 with regards to its effect on the tumor cells itself, future research and prospective studies may evaluate the specific role of miR-223 in affecting the differentiation of neutrophils, which are in turn the likely mediators and targets of miR-223 alterations. While the NEN microenvironment is a neatly regulated system and proinflammatory signaling is not generally negative [70], a feasible approach would be transferring miR-223 to (model-) organisms in NEN contexts, limiting neutrophil and Nlrp3-associated activation, possibly decreasing metastatic spread.

miR-223 has been readily discussed in the context of various cancers, e.g., affecting cellular proliferation, migration and epithelial to mesenchymal transition via the Yap/Hippo signaling in breast cancer cells [71], histamine N-methyltransferase/HER2 mediated chemoresistance [72] and apoptosis regulation via the PI3K/AKT pathway [73], amongst others [53]. While we did not find similar implications with regards to apoptosis or migration possibly to altered resistance of BON towards miR-223 signaling or/and different observational times, the persistently found activation of the Nlrp3 may possibly affect cellular survival-inducing pyroptosis [74].

Sequencing data of G1-G3 NENs revealed correlations between *NLRP3*-*PLAUR*-*CHGB*-*100A8* and *CEACAM8*/*CD66b*. miR-223 knockdown induced Nlrp3 inflammasome activation, which coincided with increased expression of *PLAUR* and decreased *CHGB*, which is represented in sequencing and in vitro data. As previously described and shown, in vitro Nlrp3 inflammasome activation affects neutrophilic immune responses, while it also correlates with CD66b expression in NEN and is somewhat predictive for disease progression. Notably, we found several patients without *NLRP3*/*PADI4* expression especially in more advanced G2/G3 NEN. Several studies have hinted to the importance of IL-1 signaling in pNET microenvironment [75], while sequencing showed the relevance of CD16, IL-1β and NF-κB signaling pathway [76], while PLAUR alterations were identified in Glioblastoma [77] and CEACAM8 in cervical small cell neuroendocrine carcinomas [78]. While our dataset is not powered to show implications on disease progression/survival, we believe these data warrant further investigation.

## 5. Conclusions

In summary, we have shown that miR-223/Nlrp3 affects neutrophils possibly via CgA; and correlated with neutrophilic CD15 expression in a small patient cohort. Adhesion marker L-selectin/CD62L was significantly downregulated in the NEN cohort, allowing for metastatic spread [51]. We accordingly performed knockdown of miR-223 in the pancreatic NEN cell line BON, finding decreased CgA intracellularly, which is proteolytically cleaved, resulting in the generation of bioactive fragments: possibly vasostatin/ catestatin [12], which may possibly affect downstream Nlrp3 activation via calcium influx, a known activator of Nlrp3 inflammasome activation [13]. miR-223 BON SN specifically altered immune cell activation in macrophages and neutrophils; and had a profound effect in direct co-culture. Whilst we found a correlation between neutrophil activation and miR-223; further research clearly warrants a look at G3 cohorts, which are known to be more immunologically active and may therefore carry a more concise profile [79]. Still G3 neuroendocrine carcinoma (NEC) are poorly differentiated and show lower expression of CgA, which may affect the Nlrp3/miR-223-related effect [80] described here. Our data hence provide initial insights into an immunoregulatory mechanism via miR-223 and CgA in NEN cells. CD15/CD62L expression on neutrophils could potentially serve as surrogate marker of cellular immunity in NEN. Future research in NEN should therefore take into account, that CgA/CgB correlate with and affect (neutrophilic) inflammation, which in NEN, in turn, was inversely correlated with miR-223 expression. While miR-223 regulation in our model increased inflammation, which may limit tumor cell survival, patient data show a regulation of miRNA between metastasis/surrounding tissue and serum; hence, miR-223 can therefore not be clearly identified as oncogenic or oncosuppressive based upon the data presented here. As ongoing research is readily concerned with tumor–immune cell interaction, looking into the relationship between CgA effectors and the direct immune microenvironment may be an important step to improve the understanding of regulatory mechanisms that limit immune response towards NEN.

## Figures and Tables

**Table 1:** Antibodies used in flow cytometry, gray indication for those antibodies stained both intra and extracellularly.

Antigen	Fluorophore	Manufacturer Prod. Nr.
CD3	SV538	Biolegend 300483
CD4	SV480	BD 746541
CD8	BB515	BD 564526
CD11b	V450	BD 560455
CD11c	AF700	Biolegend 337220
CD14	PerCPCy5.5	Biolegend 301824
CD19	PECy5	Biolegend 302210
CD25	APC/fire	Biolegend 356150
CD45	AF532	Ebioscience 58-0459-42
HLA-DR	BV570	Biolegend 307638
PD-L1	BV650	Biolegend 329740
S100A9	FITC	Biolegend 350703
CD15	PE/Cy7	Biolegend 30192325
CD16	V500	Biolegend
CD62L	PE	Biolegend 385103
CD63	APC	Biolegend 353007
CD66b	PacBlue	Biolegend 305112

**Table 2:** Human qPCR primers used for analysis of gene expression.

	Forward	Reverse
Gapdh	TTGGCTACAGCAACAGGGTG	GGGGAGATTCAGTGTGGTGG
Arg 2	GGGCCCTGAAGGCTGTAG-	AATGGAGCCACTGCC
IL1β	GGCCCTAAACAGATGAAGTGCTC	CCAGCATCTTCCTCAGCTTGTC
IL10	GGTTGCCAAGCCTTGTCTGAG	GATGACAGCGCCGTAGCC
CgA	AGAGAGGATTCCAAGGAGGC	TGATTGTTCCCCTCAGCCTTG
CgB	ATGAAGGAATGGTGACTCGC	CAGTTGTCTCTTTGTCTTTGACG
Syn	TCG GCTTTGTGAAGGTGCTGC	TCACTCTCGGTCTTGTTGGCAC
PLAUR	GGTGACGCCTTCAGCATGA	CCCACTGCGGTACTGGACAT
S100A12	CACATTCCTGTGCATTGAGG	GGTGTCAAAATGCCCCTTC
TNFa	CTGTAGCCCATGTTGTAGCAAACC	TGGCCCTTGAAGAGGACCTG
NSE	AGCCTCTACGGGCATCTATGA	TTCTCAGTCCCATCCAACTCC

## Data Availability

The raw data supporting the conclusions of this article will be made available by the authors on request. Access to patient associated data will be limited due to privacy and ethical restrictions.
